# Collaborative use of geodesign tools to support decision-making on adaptation to climate change

**DOI:** 10.1007/s11027-015-9633-4

**Published:** 2015-02-13

**Authors:** Tessa Eikelboom, Ron Janssen

**Affiliations:** 1 Institute for Environmental Studies (IVM), VU University Amsterdam, De Boelelaan 1085, 1081 HV Amsterdam, The Netherlands; 2 Spatial Information Laboratory, Department of Spatial Economics, Faculty of Economics, VU University Amsterdam, Amsterdam, The Netherlands

**Keywords:** Geodesign, Stakeholders, Spatial planning, Regional climate adaptation

## Abstract

Spatial planners around the world need to make climate change adaptation plans. Climate adaptation planning requires combining spatial information with stakeholder values. This study demonstrates the potential of geodesign tools as a mean to integrate spatial analysis with stakeholder participation in adaptation planning. The tools are interactive and provide dynamic feedback on stakeholder objectives in response to the application of spatial measures. Different rationalities formed by underlying internalized values influence the reasoning of decision-making. Four tools were developed, each tailored to different rationalities varying between a collective or individual viewpoint and analytical or political arguments. The tools were evaluated in an experiment with four groups of participants that were set around an interactive mapping device: the touch table. To study how local decision-making on adaptation can be supported, this study focuses on a specific case study in the Netherlands. In this case study, multiple different stakeholders need to make spatial decisions on land use and water management planning in response to climate change. The collaborative use of four geodesign tools was evaluated in an interactive experiment. The results show that the geodesign tools were able to integrate the engagement of stakeholders and assessment of measures. The experiment showed that decision-making on adaptation to climate change can benefit from the use of geodesign tools as long as the tool is carefully matched to the rationality that applies to the adaptation issue. Although the tools were tested to support the design of adaptation plans in a Dutch setting, the tools could be used for regional adaptation planning in other countries such as the development of regional adaptation strategies (RAS) as required by the European Union or on a national scale to support developing national adaptation plans of action (NAPAs) as initiated by the United Nations Framework Convention on Climate Change (UNFCCC) for least developed countries.

## Introduction

Spatial planners around the world need to make decisions on adaptation to climate change. This could be adaptation to droughts, floods, pests and diseases, and increasing forest fires. Dealing with these problems asks for local action. As a result, adaptation takes place at multiple spatial scales, in urban regions, mountainous regions, in both temperate as well as tropical climate zones. Recent examples are: increasing flood resilience in New York, New York, United States (Aerts et al. 2014), community-based water storage in semi-arid areas such as Ethiopia to adapt to climate change and mitigate household water shortages (Lasage et al. 2013), flood adaptation strategies for coastal cities such as Ho Chi Min City, Vietnam (Lasage et al. 2014), and developing climate resilience for alpine tourism (Wyss et al. 2014).

In practice, climate adaptation is not the main goal of regional planning (Adger et al. 2007; Ford et al. 2011). The development of adaptation plans is a complex task from both an information processing as a process point of view. Especially, because the consequences of climate change are uncertain, multiple, complex, and controversial. Adaptive spatial planning is essentially a game of mutual gain (van Buuren et al. 2013). The climate adaptation process concerns a large number of stakeholders with different backgrounds and skills for processing the information. Stakeholders are those who influence a decision, as well as those affected by it (Hemmati and Enayati 2002). The involvement of stakeholders in the planning process is increasing. Participatory approaches in environmental knowledge production are commonly propagated for their potential to enhance legitimacy and performance of decision-making processes (Hage et al. 2010). However, the involvement of stakeholders is generally costly and time consuming (McIntosh et al. 2008). Careful preparation work on planning activities is required for successful participation. Furthermore, participation must be organized as an explorative process to create operational collaboration (Celino 2011). Collaborative planning is an interactive process of consensus building using stakeholder and public involvement. Margerum (2002) stressed out the importance of a pragmatic approach and a skilled facilitator. Another barrier for collaborative planning is the availability of data and methods to develop, assess, and select measures (Moser and Ekstrom 2010).

Improvements of the current organization of the spatial planning system are desirable to enable the realization of climate adaptation (van Buuren et al. 2013) as adaptation plans are largely underdeveloped (Preston et al. 2011). Improved climate-related decision-making requires the acknowledgement that information may be scientifically relevant without being decision-relevant. The tools and scientific information that scientists consider as simple and useful are not always perceived that way by practitioners (Beunen and Opdam 2011; Kirchhoff et al. 2013). In addition, different actors perceive the usefulness of scientific information differently. Decision support characterized by one-way communication and a focus on products as opposed to process has been demonstrated to be ineffective (Weaver et al. 2013).

Spatial information plays a key role in the design of adaptation strategies as climate change has spatial impacts (e.g., Wilson 2006). In addition, the adaptation strategies themselves are spatial as they involve the spatial allocation of measures. The spatial information includes multiple layers of information ranging from detailed technical information to more general and sometimes qualitative information on development paths for a region. A geographic information system (GIS) is an indispensable tool for planners to design and visualize the effects of their decisions. The capabilities of a GIS can help to educate stakeholders about potential impacts of certain decisions on their objectives (Schatz et al. 2013).

Multiple tools already exist that support the adaptation development process such as decision support systems (DSS) and planning support systems (PSS) (Geertman et al. 2013). Three recent examples are LandCaRe DSS, a vulnerability assessment model (Giupponi et al. 2013); a decision support system for urban climate change adaptation named SUDPLAN (Gidhagen et al. 2013); and a DSS for identifying and exploring the potential of adaptations strategies to cope with flood risk (Ceccato et al. 2011). In addition, more general planning tools exist that do not explicitly refer to climate change, but which can be suitable for adaptation such as the “Online What If” planning support system (Pettit et al. 2013). Wenkel et al. (2013) found from stakeholder workshops that for decision support systems in climate change science, tools should interactively communicate state-of-the-art knowledge, provide easy-to-use regional climate information, and enable simulations of adaptation options.

Geodesign tools combine geography with design by providing stakeholders with tools that support evaluation of design alternatives (Flaxman 2010). The integration between spatial data, collaboration, and decision-making in geodesign tools poses several challenges. Firstly, there is the social dimension of the problem as the technology must be perceived useful to be accepted. Secondly, emotional factors such as satisfaction and commitment to the process can play a role. Thirdly, there is the organizational dimension of the problem (Antunes et al. 2013). The behavior of stakeholders can differ based on underlying internalized values that form the foundation of a rationality or scheme of reasoning. Different rationalities can result in different spatial designs based on the same empirical observations. Carton (2007) described two types of classifications to distinguish between rationalities, the viewpoint of actors and the type of reasoning. The division of viewpoint is between collective rationality, where the stakeholders have the willingness to engage based on a common ground, and individual rationality, which is about strategic behavior of stakeholders to strive for their own benefit. The collective viewpoint builds on the concept of communicative rationality, and the individual approach focuses on the behavior of individual actors in safeguarding their values. The classification of rationality by the nature of arguments is in analytical and political reasoning. The reasoning behind decision-making can be more analytical, where the focus is on objectives, or political which relies on the creation of benefits from stakeholder perspective. Political distinguishes itself by focusing on stakeholders separately, whereas the collective view combines stakeholders. Developing usable tools requires systematic research to better understand the science–practice interface. Experimentation with new scientific tools in practice helps to observe how practitioners respond and how the tools affect the social process (Opdam et al. 2013).

In this study, a case study was used to show how geodesign tools that are tailored to different rationalities support the development of local adaptation plans. These tools support the identification of adaptation measures by providing feedback on different objectives and stakeholders interactively. The tools were designed based on different rationalities as it was expected that the collaborative use of tools differs between adaptation issues and involved stakeholders.

**Fig. 1:**
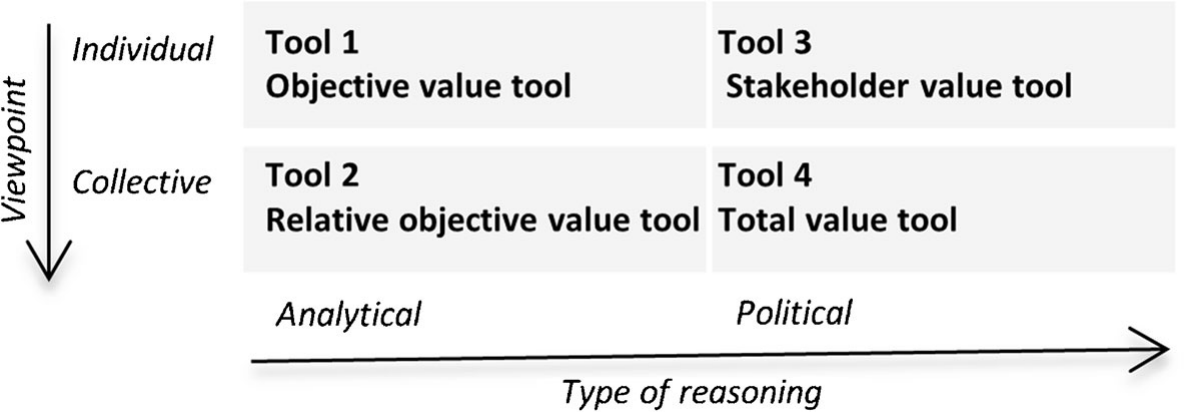
Classification of four geodesign tools by rationality (based on Carton 2007)

Four geodesign tools were developed according to the classification of rationalities (Fig. 1). The tools are named the objective value tool, the relative objective value tool, the stakeholder value tool, and the total value tool. Each tool is expected to suit specific adaptation issues based on the type of stakeholders that are involved, the adaptation development stage of the planning process, and the regional characteristics of the area and problem itself. The analytical and individual tool is expected to be useful for adaptation issues that involve few or collaborative stakeholders and more quantifiable issues such as crop change, whereas the collective- and political-based tools provide information from stakeholder perspectives. The tools were evaluated in an experimental setting to study how the tools and the associated rationalities influence the decisions of the planners and researchers in designing land use and water management changes in response to climate change.

## Material and methods

To study how regional decision-making on adaptation can be supported, this study focuses on a specific case study in the Netherlands. In this case study, multiple stakeholders need to make spatial decisions on land use and water management planning. The collaborative use of four geodesign tools was evaluated in an interactive experiment with two groups of researchers and two groups of planners. The participants were asked to use each tool to design spatial measures, in the form of land use and water management changes, to improve the value of one objective while at the same time minimize the decrease in the other two objectives. The designs were compared by the amount of measures initiated by the tool, the correspondence of the measures with tool information, and the change in objective values. In addition, the communication and behavior of the participants was observed, and the perception of the participants was assessed in a survey containing 70 statements. The next sections illustrate the format of the experiment, provide tool descriptions, and describe how the results were compared.

### Experiment

Two experimental sessions were organized to evaluate the geodesign tools in a controlled setting. The advantages of an experiment in a controlled setting compared with a planning process are (1) time taken to introduce new tools to stakeholders, (2) the opportunity to ask them for feedback, and (3) allows for comparative studies by applying multiple tools in a row for the same task. The tools were integrated in an interactive decision support system. An interactive mapping device, Microsoft Surface 2.0 Touch Table, was used as the communicative platform, similar to previous studies (Alexander et al. 2012; Arciniegas et al. 2012; Janssen et al. 2014). The advantages of a touch table, such as learning by doing, availability of a geo-spatial database, and intuitive control, have been described in several studies (Arciniegas et al. 2011; Eikelboom and Janssen 2015; Pelzer et al. 2013).

Two simultaneous groups worked on separate devices to compare results. The first experiment was organized for researchers, and the second experiment was organized for regional planners. The researchers were associated to environmental sciences related to peat meadow areas. The participants of the planners’ session were from Dutch provincial authorities and water boards involved in planning in peat meadow regions. Both groups are involved in peat meadow area but not in this particular study area. In total, there were 14 participants. Before and after the experiment, the participants filled in a survey. The opinion of the participants was assessed by 70 statements using a 5-point Likert scale. The categories of the survey were (1) personal characteristics, (2) decision context, (3) role of climate change, (4) information needs, (5) experience, (6) experiment feedback, and (7) tool feedback. The number of participants was limited due to the time effort requested from the participants. The aim of this study was to evaluate the group decision processes with real stakeholders, but researchers and planners in the field of peat meadow areas have limited availability.

**Fig. 2:**
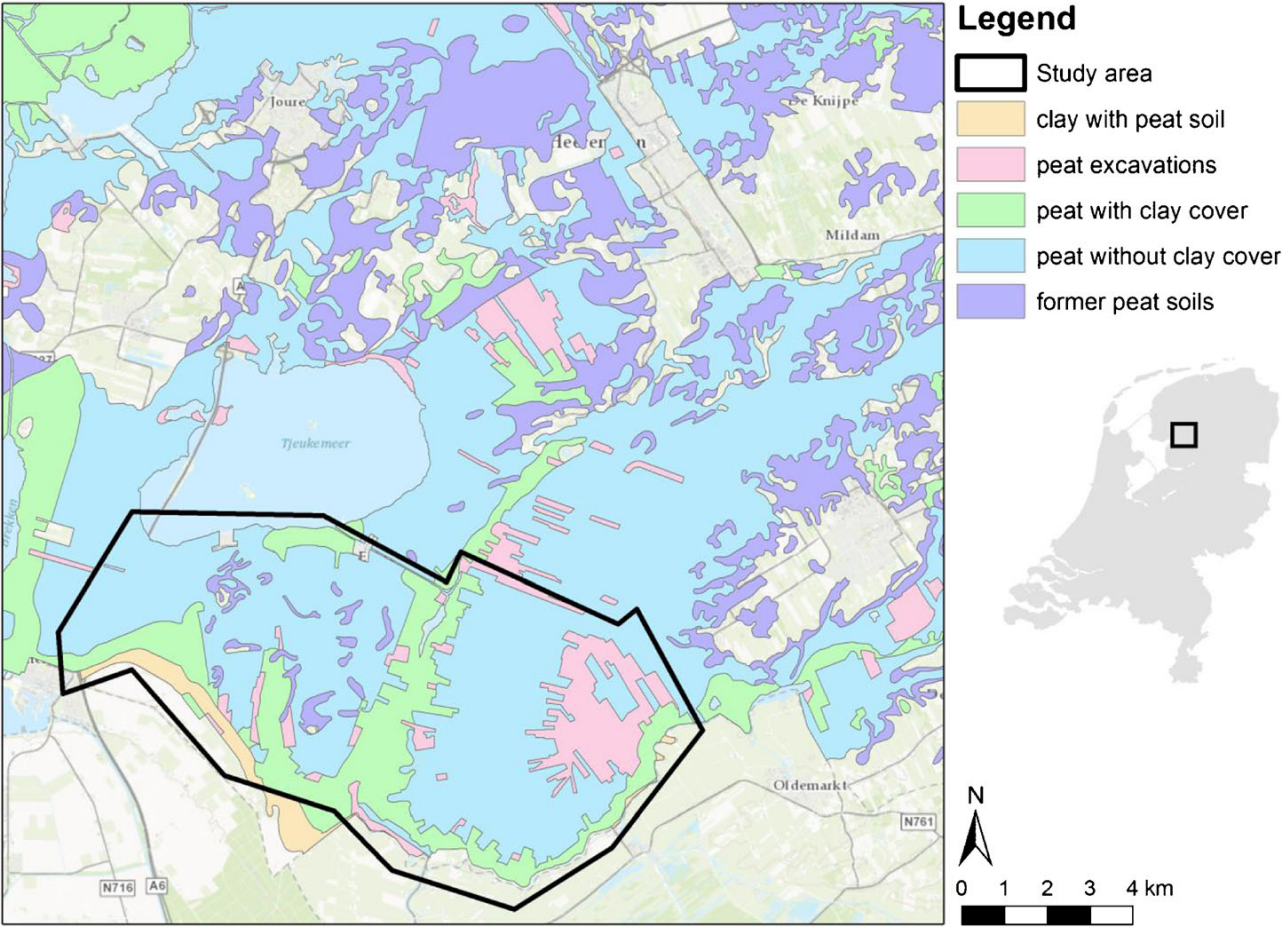
Location of the peat meadow study area

The study area was a peat meadow area of about 50 km^2^ in the northern part of the Netherlands (Fig. 2). The Province and Water Board of Friesland have decided to develop a long-term adaptation strategy for the peat meadow areas of the province. Primary activities in this region are highly productive dairy (*Bos*) farming, nature conservation, recreation, and housing. The region is currently mainly used for commercial dairy farming but is also important for its high natural, cultural, and historical values. Important problems in the region are soil subsidence causing damage to buildings and infrastructure, deterioration of landscape values, inefficient water management, poor water quality, and the changing perspectives for dairy farming (Janssen et al. 2014). More details on application of the tools in peat meadow areas in the Netherlands can be found in Brouns et al. (2014) and Janssen et al. (2014).

Measures are needed to be taken to address the problems of soil subsidence without too much damage to agriculture. Spatial measures such as land use change and changes in water management were found to be relevant adaptation measures. Agriculture, soil subsidence, and nature were identified by the stakeholders as the three main objectives, and their value was dependent on water level and land use. The effects of climate change were accounted for in the water level and in soil subsidence, and a change in these variables resulted in a change of the objective values. Climate change was incorporated by using the W+ scenario from the Royal Netherlands Meteorological Institute (KNMI) and predicts a temperature increase of 2 ° and a modified atmospheric circulation, resulting in drier summers (van den Hurk et al. 2006). The change in subsidence rates was assumed to increase at a rate of 1.5 (Brouns et al. 2014).

**Fig. 3:**
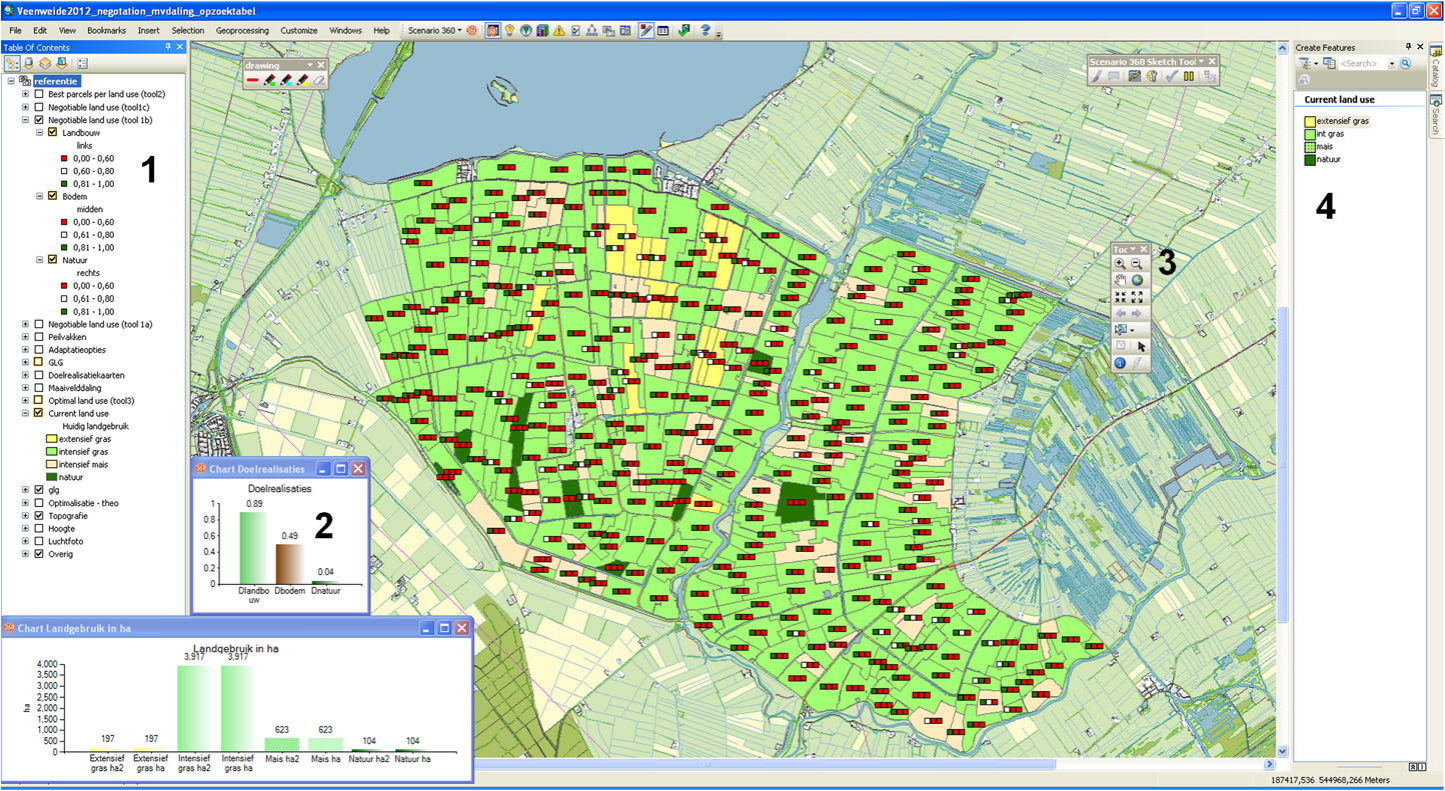
Graphical user interface of a geodesign tool with the (*1*) map library, (*2*) bar charts of land use surface area and average objective values, (*3*) navigation toolbar and (*4*) design toolbar for applying measures. The map shows the start situation. The traffic lights represent the values of three objectives where *red* means low, *white* medium and *green* is high

The tools were constructed with Community Viz software version 4.3 (http://placeways.com/communityviz, last accessed December 2014). Figure 3 shows the graphical user interface, which was used by the participants during the experiment. The interface enabled real-time analysis of applied measures, but did not include sophisticated physical, social, or economic models. Instead, the tools used an expert-based multi-criteria analysis to determine the effects of measures on multiple objectives. In addition, the values were aggregated to the level of parcels.

The experimental sessions contained four rounds of 1 h each. The same assignment was used for each group and each tool. The assignment was to improve the value of one objective (soil subsidence) while at the same time minimize the decrease in the other two objectives (agriculture and nature). To achieve this, the groups could change the land use or water management of the region. The changes in land use should be done in such a way that the totals for each land use stayed the same. In each round, the participants applied measures on the level of parcels. This resulted in a set of 16 maps with different spatial designs. During each round, participants experimented with applying measures as they were also able to undo measures or to adjust the measures based on tool feedback. The amount of potential designs was high as the possible measures could vary between three types of land uses and ten classes of water level change. The designs can be dominated by a single participant. On the other hand, the suggestions of a single person could as well be easily turned back by the other participants. For each group, the geodesign tools were used by the same group of participants to ensure comparability between tools.

### Tool description

Four geodesign tools were developed tailored to different rationalities. This means the tools were divided into individual or collective viewpoint and analytical or political reasoning. Political reasoning differs from the collective viewpoint as political treats each stakeholder separately, whereas collective concerns the interests of multiple (Carton 2007). The individual tools strive for strategic decisions. The analytical tools present objective values. The collective tools combined stakeholder values using weighting factors to provide a common ground for stakeholders by suggesting locations for action. The political tools stimulate negotiation by showing preferred changes and can be used to make trade-off decision. Each tool fits one unique class of combination from the division of rationality in individual and collective reasoning, and the split in arguments for analytical or political tool use. The tools are named (1) the objective value tool, (2) the relative objective value tool, (3) the stakeholder value tool, and (4) the total value tool. The objective values were derived from expert-based look-up tables that used land use and water level as input parameters.

**Fig. 4:**
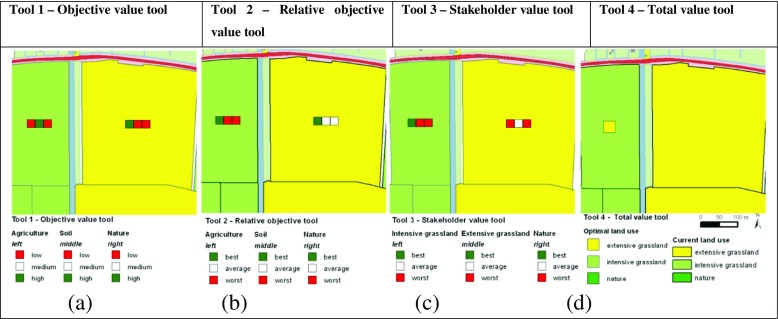
Visualization of four geodesign tools for two parcels that show: **a** absolute objective values, **b** relative objective values, **c** relative scoring of each land use based on stakeholder weights, and **d** the land use change that is best for all stakeholders

The objective value tool was designed to support a decision-making process that is focused on analyzing objectives at the individual level. For each parcel, the absolute objective values are shown. These values change when water level or land use is changed. Each objective is presented as a traffic light symbol of three classes where red is low, white is average, and green is a high value. In Fig. 4a, the left parcel is scoring high for the objective soil and low for agriculture and nature. The right parcel is scoring high for agriculture but low for the other two objectives. The difference between the objective values is caused by the difference in land use and water level.

Conversely, the relative objective value tool aimed to support collective planning. The relative objective tool shows relative values instead of absolute values. The tool indicates the status of the parcel in relation to the values of the other parcels. In this way, relative differences can be observed even if the whole area has low values for an objective. The tool can be used to easily find parcels with high and low combinations which can be subject to change. This means that traffic lights are green if the objective value is high compared to other parcels or red if the value is low compared to other parcels. If the height of a value is average, then the traffic light is white. A fixed border of the percentage of best and worst objective values is set. In this example, the threshold was set at 20 %. The 20 % best are given a green light, the 20 % worst are given a red light, and the 60 % in between are white. When the objective value of a parcel changes due to water level of land use change, the ranking is updated and the distribution of red and green traffic lights changes. Although the objective value tool indicates that the agricultural value of the intensive grassland of the left parcel in Fig. 4a is low, this value is still relatively high compared to other parcels in the area based on Fig. 4b. Similarly, the agricultural value of the extensive grassland is relatively high.

As planning involves multiple stakeholders, a tool that combines objectives by weighting according to individual preferences is the stakeholder value tool. This tool contains the potential objective values for each land use type. The left traffic light is intensive grassland, extensive grassland in the middle, and the right traffic light represents nature. Weights for intensive grasslands were set 1.0 for agriculture, 0.0 for soil, and 0.0 for nature. Weights for extensive grasslands were set 0.5 for agriculture, 0.25 for soil, and 0.25 for nature. Weights for nature were set 0.0 for agriculture, 0.25 for soil, and 0.75 for nature. The weights were set a priori based on expert knowledge. The participants were informed about the reasoning behind the weights and were given the opportunity to change the weights during the experiment, but they did not use this opportunity.

The traffic lights show whether a land use type potentially has a relative high value, a relative low value, or in between. The traffic lights are static and independent of the current land use. The traffic lights that represent a land use type with a high potential total value is green. This tool stimulated participants to search for favorable swaps from red to green. If for example, the current land use is intensive grassland and this light is red, but the light of extensive grassland is green, a land use change from intensive to extensive grassland is favorable. Figure 4c shows for the left parcel that intensive grassland is the best scoring land use according to the current land use. On the other hand, the right parcel is extensive grassland but extensive grassland is classified as being one of the worst land uses for this parcel.

The total value tool used weighting factors, defined by stakeholders, to combine objectives in collective values. It has a different map lay out as it only presents one single traffic light for each parcel instead of three (Fig. 4d). The background color of each parcel again shows current land use. The traffic light shows the land use that results in the largest total value. If the current land use is the same as the best land use, no traffic light is shown. Figure 4d shows that for the intensive grassland, a change to extensive grassland would lead to the highest total value.

The properties of the tools correspond to the demands of Pouwels et al. (2011) who purposed that tools should be built on interactions between functions, encourage interaction, allow incorporation of local knowledge, and generate output in the form of a map that shows where conflict areas and opportunities are located. More detailed descriptions of the tools can be found in Eikelboom and Janssen (2015).

### Analysis of results

The influence of geodesign tools on the use of tool information were analyzed from the designs on the maps and from observations of the communication in the groups. The designs were evaluated by, the type of measures, the spatial distribution, the number of changes, the number of changes that correspond with tool information, and the change in objective values. The last three were assessed numerically. The correspondence to tool information was defined as the number of parcels that were changed that fitted the information provided by the color of the traffic light in that parcel. The percentages were expressed in relation to the total number of parcels in the area. A parcel that was changed in multiple steps is not counted separately. Only the final change map compared to the original map was used to calculate the number of changed parcels compared the total number of parcels.

The objective values were expressed as absolute means for the whole area. The number of measures applied by the participants was used as an indicator for the functioning of the tool, the stimulating effect, and the perceived usefulness. The assumption was that a more difficult tool would use more time to be interpreted which would cause less time to be spent on allocating measures. In addition, the number of changes made with the tool provides an indication for the extent to which the tool stimulates change. The communicative potential of the tools was derived from the correspondence of the measures with tool information as that indicates whether the information as indicated by the tool was used for the decision. Furthermore, differences between the groups in using tool information were derived from observations and recordings. The responses to statements of the survey were averaged to discover statements with high impact and to compare the responses of planners and researchers.

## Results and discussion

First, the quantitative results were provided as the percentage of changed parcels, the percentage of changes in accordance with tool information, and the mean objective values. Next, the results of comparing the sessions of the four different groups for the objective value tool were described on the basis of the four designs. Third, the observations of the experiment were described for each type of tool including an example of measures applied with each tool. Finally, the feedback of the participants was described.

### Comparison of the spatial designs

The amount of measures from each group and for the geodesign tools is shown in Table 1. The use of the objective value tool by the first group of researchers resulted in the most changes (66 %). The other groups changed between 16 and 19 % of the parcels with the objective value tool. The relative objective tool induced less changes for the second group of researchers and the second group of planners. Again, the first group of researchers applied most changes. Also, for the stakeholder value tool, the first group of the researchers applied most changes, though the amount of changes was only 7 %, which corresponds to 25 parcels. The mean percentages demonstrate that the objective value tool induced most changes and the stakeholder value tool the fewest. Both tools were classified as individual, which in view of rationalities indicated that more changes resulted from the analytical tools compared to the political tools. The observations showed that participants made less use of the tools when they felt the tool was less useful. This finding corresponds to the theory of the Technological Acceptance Model (TAM) (Davis 1985; Dias and Beinat 2009).

**Table 1:** The amount of measures from each group and for the geodesign tools expressed as the percentage of the area

Geodesign tool	Researchers 1 (%)	Researchers 2 (%)	Planners 1 (%)	Planners 2 (%)	Mean (%)
(1) Objective value tool	66	16	18	19	30
(2) Relative objective value tool	21	8	18	2	12
(3) Stakeholder value tool	7	2	5	2	4
(4) Total value tool	10	19	6	6	10

Table 2 shows that the measures designed with support of the objective value tool and the total value tool were more in correspondence with tool information compared to the other tools. The rates of the objective value tool are above 70 % for all four groups. The correspondence of the relative objective value tool is poorest (57 %). This suggests that many changes designed with support of this tool were not based on tool information. The correspondence of the relative objective value tool is lower than the objective value tool and the total value tool. In general, suggestions from the total value tool were followed which is reflected in a mean performance rate of 91 % correctness.

**Table 2:** Correspondence rate of measures with tool information from each group and for the geodesign tools

Tool	Researchers 1 (%)	Researchers 2 (%)	Planners 1 (%)	Planners 2 (%)	Mean (%)
(1) Objective value tool	81	70	91	91	83
(2) Relative objective value tool	49	60	68	50	57
(3) Stakeholder value tool	73	100	78	50	75
(4) Total value tool	94	99	100	72	91

Linking the results does not show a relation between the amount of changes and the correspondence of the changes. As an example, “Researchers 1” applied many changes according to tool information with the objective value tool as they changed 66 % (Table 1) and of these parcels 81 % (Table 2) were changed according to the tool information. Another finding is that despite changing a high number of parcels simultaneously, the correctness of the changes is still high. There are also examples with only a low percentage of change but with a high performance as well as examples of few changes with low performance. Both tables show a high variability between groups and tools.

Furthermore, the average value for each objective was calculated as the area weighted sum of the values for each parcel. The values suggest that it was fairly easy to keep the value for agriculture above 0.80. In the current situation, agriculture has a mean value of 0.85, soil subsidence is 0.49, and nature has a value of 0.08. The objective value tool supported the first group of planners to accomplish the highest score for soil of 0.61 and a score of 0.25 for nature as they combined extensive agriculture with high water levels. The same group again produced the highest value for soil (0.54) with use of the relative objective value tool. The political tools, stakeholder value tool, and total value tool, did not or only minor result in water level changes, though the water level highly influences objective values. Consequently, no increases in objective values were achieved with the stakeholder value tool.

### Comparison between groups

**Fig. 5:**
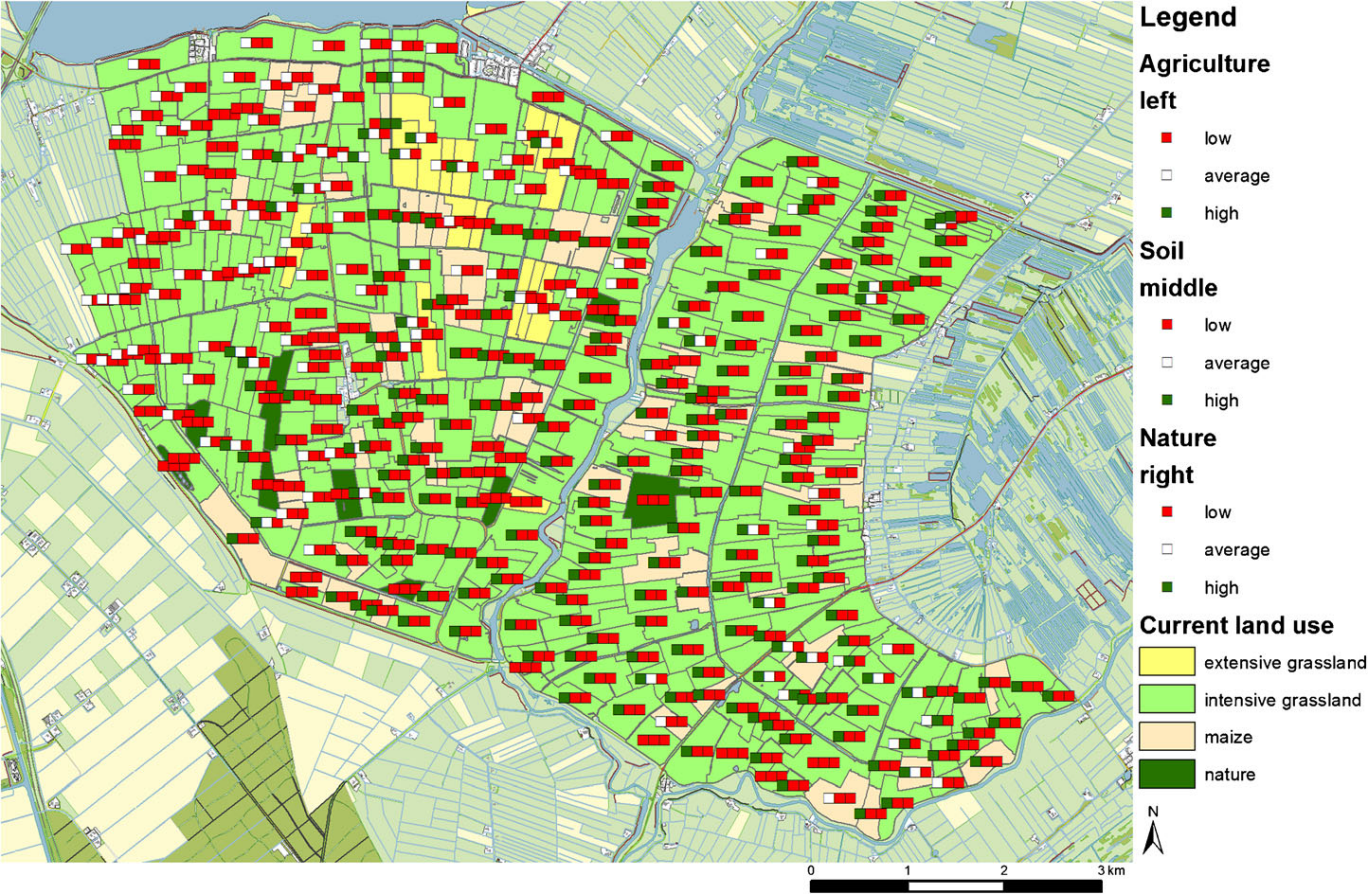
Original land use map with objective values of start situation

**Fig. 6:**
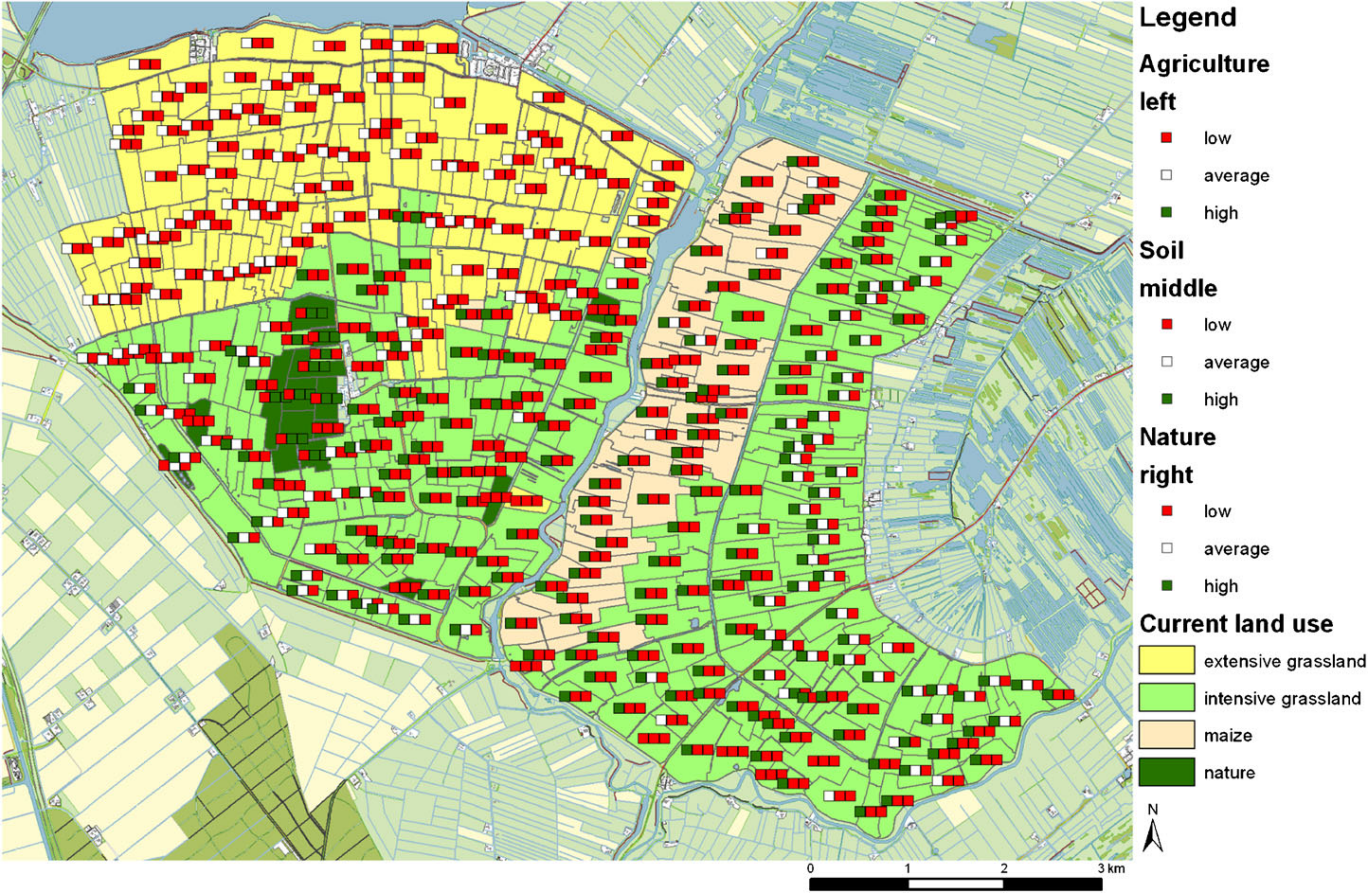
Spatial design of the first group of researchers. The traffic lights show the updated objective values

**Fig. 7:**
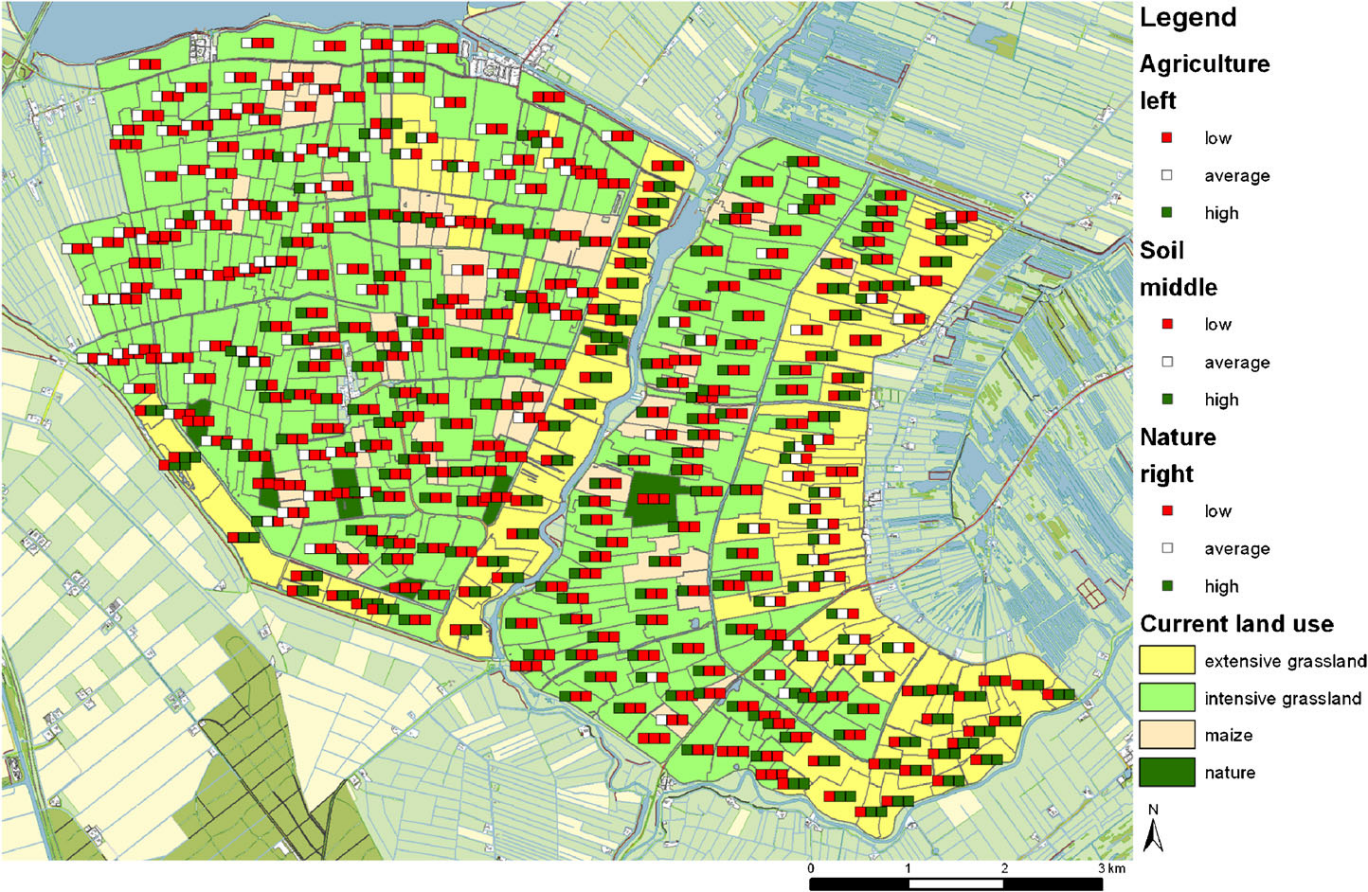
Spatial design of the first group of planners. The traffic lights show the updated objective values

The comparison of the maps supported with the objective value tool revealed different designs for each group of participants without consistent differentiation between researchers and planners. Figure 5 shows the original situation that was used to start the design session. The first group of researchers introduced a large area of extensive grassland (Fig. 6) and decided also to raise water levels on multiple parcels. In addition, the participants decided to move the maize parcels along the right side of a river as it was observed from the soil map that these were clay soils. Next, the second group of researchers noted that nature was fragmented and decided to cluster nature and moved it northeast. This group also focused on land use changes such as moving maize. The group also added different small groups of extensive grassland. Subsequently, the first group of planners introduced a buffer of extensive grasslands in the east as this area is adjacent to a large nature area that is located outside the study area (Fig. 7). However, they did not combine this measure with raising the water level. This group also focused on buffers of extensive grasslands and combined this with changes in water level. Furthermore, only a few nature parcels were removed. The group shifted a lot of parcels from scoring high on agriculture and low on soil and nature into low for agriculture and high on soil and nature. Finally, the second group of planners also introduced a large area of extensive grasslands and two smaller clusters. The group increased the water level for some deep wells based on the elevation map. No changes were made for maize or nature. Similarities in the designs can be observed for the first group of researchers and the second group of planners as well as for the second group of researchers and the first group of planners.

Explanations for the different designs between groups and tools were found in variations of the observations. The first difference was observed in the time spent on tool interpretation. Some groups used more time to discuss what could be observed from the tool. Secondly, the duration of negotiating the allocation of measures varied. In some groups, less negotiation preceded the design of measures. Related to this, the second observed difference was that some groups applied large clusters of measures, whereas others changed individual patches. The different designs changed the objective values as observed from the traffic lights. The objective values also differ due to changes in water level, which cannot directly be observed from the figures in this paper but the water level maps were available in the application used during the experiment. Despite the design of different maps, the types of discussions were similar for the four groups. The same logic towards deciding on the type of measures was restated such as moving of the maize parcels, create higher water levels for intensive grasslands or combine the high water levels with extensive grassland, and cluster nature areas together. Only the spatial allocation of these measures differed for the four groups.

### Comparison between tools

**Fig. 8:**
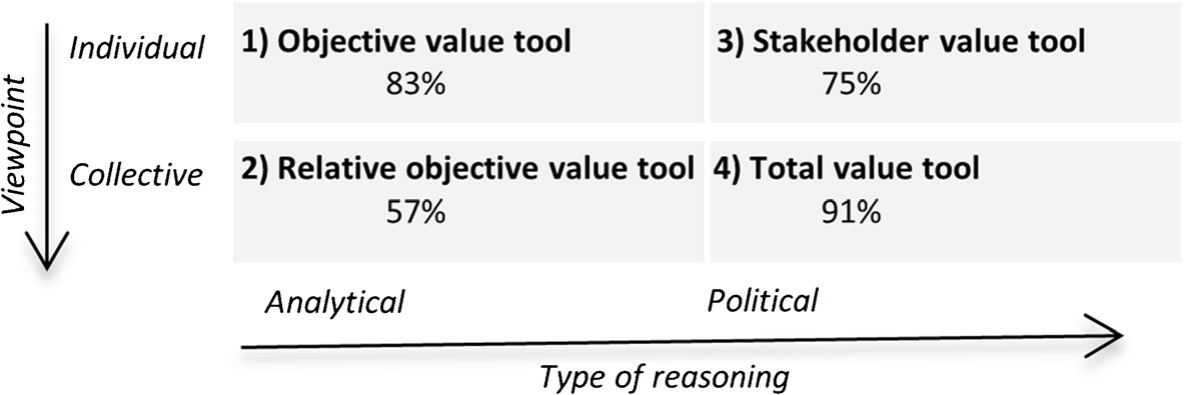
Percentage of the applied measures in correspondence with tool information

The previous section described differences between the participants for a single tool. Similar to the results of the objective value tool, the other tools also showed similarities in the type of applied measures. Each tool supported the group decision process differently, varying between collective, individual, analytical, or political rationality (Fig. 8). A review of how performance of planning support systems is reported (Te Brömmelstroet 2012) showed that most studies hypothesize about which dimensions were improved but did not report on measuring this increase. The designs in this study were judged quantitatively by the amount of changes and the correspondence of the changes to tool information. The resulting percentages suggested that some tools encouraged less measures. In addition, the performance of the measures indicated that some tools induced more changes that were not in correspondence with tool information. The performance of the measures with the tool tailored to collective and analytical rationality was poorest. This was either caused by the difficulty of tool information or by the use of expert knowledge to decide on measures. This section describes the typical observations for each tool.

**Fig. 9:**
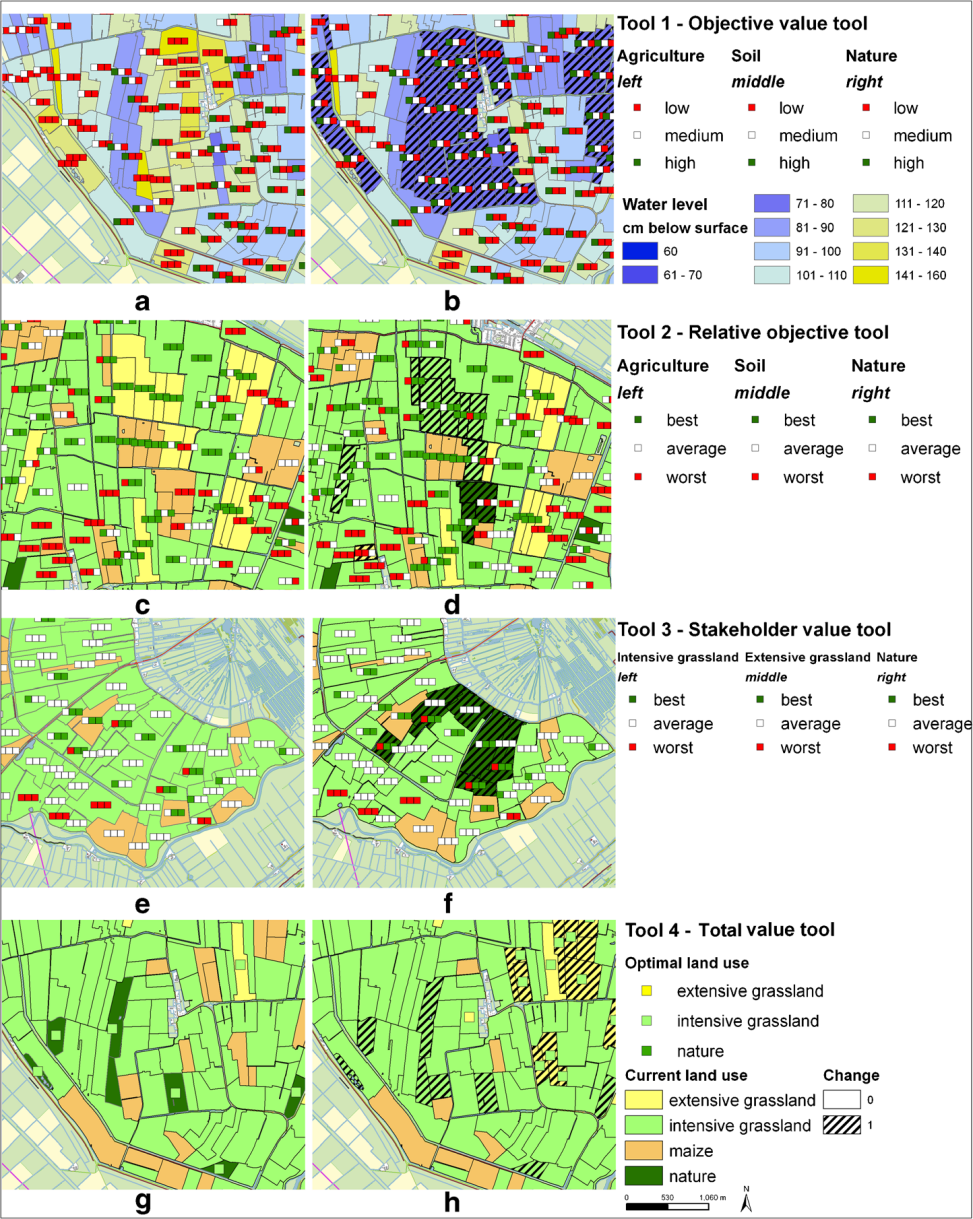
Examples of measures applied during the experiment with the original situation on the left and the changes on the right for each tool

The following examples of measures illustrate the use of each tool (Fig. 9):

1. Objective value tool—raising the water level improved the objective agriculture and soil.
2. Relative objective value tool—the change of extensive to intensive grasslands was unfavorable.
3. Stakeholder value tool—allocation of nature to suited parcels with some connecting parcels.
4. Total value tool—nature was changed to intensive grassland according to tool information.

The objective value tool corresponded to individual and analytical rationality. The use of the objective value tool resulted in changing large clusters of parcels where the focus was on the objective value of soil. Only little time was spent on discussing tool interpretation and negotiating the allocation of measures. In the example of Fig. 9, the participants decided to raise the water level for parcels with three red lights. Comparison of the new situation (Fig. 9b) with the original (Fig. 9a) reveals that the higher water levels were beneficial for both agriculture and soil, but the objective value of nature was unchanged. Apparently, the original water level was too dry for agriculture, soil and nature. The new situation was still too dry for nature.

The best and worst values obtained with the relative objective value tool were suited for more collective behavior but was analytical, because it describes three separate objectives. Application of the relative objective tool resulted in changes for single parcels with a focus on relative low agricultural values. Much time was spent on discussing tool interpretation and negotiating the allocation of measures. The examples for the second tool showed that tool information was not always the main driver for changes. The participants changed some extensive grassland into intensive grasslands. The traffic lights indicated a relative high value for the three objectives. This means no suggestion for applying land use change is initiated by the tool. However, the participants decided to investigate whether the objective values would decrease when changing land use to intensive grassland. This is an example of experimental use of the tool. After changing to intensive grasslands, the traffic lights indicated that for the most left parcels, the relative objective value for agriculture was not within the best anymore. The change of the traffic lights due to the application of measures was found to be confusing. The tool calculated a new subset of best and worst objective values. Next, participants changed two maize parcels to nature. One of the maize parcels has a green traffic light for the objective nature, and the other two maize parcels are within the worst scoring parcels for agriculture (Fig. 9c). It is therefore reasonable to change these parcels to another land use type such as nature as performed by the participants (Fig. 9d). Also, a single maize parcel was changed to extensive grassland. The maize parcel in the lower left corner has a relative low value for the three objectives. For such a parcel, the tool suggests that any change could be beneficial. Changing land use to extensive grassland improved the relative score for nature. From the observations of the conversations, it was found that this tool was less intuitive and resulted in more discussion on the response of the tool. This is reflected in more random changes to discover how the traffic lights respond in comparison to their expectations.

The stakeholder value tool presents the interests of individual stakeholders and couples to political use. The use of the stakeholder value tool mainly led to single changes, but these changes concentrated on parcels with a combination of red and green traffic lights. In the study area, only red, green, green parcels existed. The other parcels had either three blank, three red, or three green traffic lights. Again, a lot of time was spent on discussing tool interpretation and on negotiation of allocating measures. The traffic lights suggested that intensive grassland was not the best scoring land use. In contrast, for some of these parcels, the right traffic light indicated that nature is a potential high scoring land use. The participants used this explicit information to decide to change the parcels from intensive grasslands to nature (Fig. 9f).

The total value tool used a summation, which implies a collective context, and used weighting of the objective values, which refers to political reasoning. The application of the tool showed that little time was spent on discussing tool interpretation, whereas more time was spent on negotiation of allocating measures. The designs were strategic and in clusters. The participants used the tool to look for sets of parcels where one suggests a change from A to B and the other suggests the reverse. Once the sets were finished, no further measures were taken as the assignment was to preserve the amount of hectares per land use. In exchanging land use, the participants mainly designed clusters of changes. In addition to changes suggested by the tools, participants also made their own changes based on local knowledge or to test the response of the tool. This is reflected in the results of Fig. 9h. Apart from the tool, the participants also decided to add extensive grasslands. However, the tool showed that the total value was highest for intensive grasslands.

For both analytical tools, the participants mainly based their decision on a single traffic light. The relative objective value tool, collective and analytical, and the stakeholder value tool, individual and political, were subject to much more discussion about the interpretation of the tools which led to less changes. This was especially observed for the second group of planners.

### Feedback on the tools from the participants

Before and after the experiment, the participants filled in a survey. The survey included 70 statements assessing (1) personal characteristics, (2) decision context, (3) role of climate change, (4) information needs, (5) experience, (6) experiment feedback, and (7) tool feedback. A selection of the five strongest statements was made (both positive and negative deviations). These five statements were: (1) the tools support a first identification of measures, (2) the effects of changes visible in bar charts is useful, (3) usage of indicative values when no exact values are known is useful, (4) the tools supports finding measures to reduce soil subsidence, and (5) exchanging land use was easy.

The decision context and role of climate change were studied to gain information on the institutional settings of the study area. Statements on the decision context revealed that the complexity of the decision process of soil subsidence does not so much relate to contradictory objectives, stakeholder disagreements, politics, the long time horizon, or history. On the contrary, the statements towards climate change indicated the need to reduce uncertainties, create consensus on the occurrence of effects, and identify what types of adaptation measures exist, the consequences, their timing, and location.

In terms of information needs, the participants prefer to receive information about breaking points instead of trends. Currently, prevailingly scenarios were used to illustrate the effects of climate change, however information is requested on what situation can no longer be maintained. Moreover, they like to see the differences compared to the current situation. The participants highly indicate their need on information on consequences of climate change (64 %), possible measures (79 %), effectiveness of measures (71 %), and cost-benefits of measures (71 %). To deal with the problems in this study, multi-criteria and cost/benefit approaches were preferred above design or expert-based approaches.

Although, the planners were less experienced with the use of a Touch Table compared to the researchers, the four groups indicated that the tools were easy to use. The average rate of the experiment on a scale from 1 to 10 was 7.5. The participants stated they would recommend the methodological concept to others. Furthermore, the experiment contributed positively to extending their knowledge on the possibilities of interactive spatial support tools (mean rate of 3.93 on a 5-point scale).

Finally, participants were asked to provide feedback on the tools. The evaluation of the four different tools showed a preference for the objective value tool (78.6 %) and the total value tool (35.7 %) compared to 7.1 % for both the relative objective value tool as well as the stakeholder value tool. As stated by one of the participants: “The analytical tools show the effects of measures on single objectives, whereas the other tools force integration.” Participants’ comments on the tools also provided suggestions for tool requirements, such as the need for a professional operator to control the use of a tool during a session. Furthermore, it was suggested to provide more explanations on how the traffic lights change differently for each type of tool. Also, information was requested about the stages of the planning process in which the tools can best be applied. In addition, consensus was needed between stakeholders on the weighting of values that was used in some of the tools. Some mentioned drawbacks of the tools were the time effort for applying the tools on new study areas, the fact that decision-making also depends on aspects that were not included in the tools such as politics and finances, and that the results were highly dependent on the formulas and models used in the tools. Comments on the tools also provided suggestions for tool improvements. Currently, the valuation does not incorporate spatial adjacencies of similar land uses in adjacent parcels. Suggestions on the appearance of the tools were to fade out parcels that are not subject to change and combine the tools with 3D visualizations and photos. Ideas on the content were to only show priority areas, to include a filter to select parcels with similar characteristics at once, and to include scenarios that show the influence of a single measure for the whole area such as increasing summer water levels.

Furthermore, the statements were compared between planners and researchers. Table 3 shows the statements that had different responses for each group. Interestingly, planners agreed more on the statement that the maps in the touch table highly influenced their decisions. They also were more positive about that applying changes on the level of parcels was useful and that the tools supported the tasks.

**Table 3:** Different responses to statements between researchers and planners (5-point scale)

Statements	Mean researchers	Mean planners
Experience with touch table	3.10	1.50
Information availability of the touch table	2.70	3.75
Better use a touch table instead of paper maps	4.00	3.00
The maps in the touch table highly influenced the decisions	3.10	5.00
The information in the touch table was synoptic	3.30	4.25
The applied changes on the level of parcels was useful	3.30	4.50
The tools fit the spatial level of the problems	2.56	1.75
The tool support the tasks	3.44	4.25
Climate change is a large problem for the region	4.00	3.25

The selection of a tool should depend on the type of rationality that fits the adaptation problem. This is influenced by the area-specific characteristics of the adaptation issue and the tasks involved in designing a plan for the region. These tasks vary during the process as each adaptation development stage has different tasks (Eikelboom and Janssen 2013). The collective tools suit a communicative approach, whereas the tools based on individual rationality focused on the behavior of individual actors in safeguarding their values. Next, there is the difference between the need for an analytical approach of the effectiveness of measures or a political approach that focuses on how stakeholders prioritize measures. As an example, a climate change-driven decision for changing crop type due to, for instance, drought or diseases by a single farmer can be defined as an individual and analytical decision. The objective value tool can support this decision by providing insight into the influence of crop changes on single objectives. On the contrary, a regional dike reinforcement project is much more a collective endeavor. In this case, the relative objective value tool can provide support by visualizing the best and worst performing areas. The allocation of flood prone areas is certainly a political example of adaptation as the measure solves a larger scale problem at the expense of local interests. The stakeholder value tool can provide support by visualizing changes for each stakeholder separately as it uses weighting factors according to stakeholder preferences. In a planning situation where there is agreement on the relative importance of the different objectives, the total value tool is a tool that gives fast and specific suggestions on optimal land use changes.

## Conclusions

Decision support to develop viable climate change adaptation strategies encompasses a wide range of measures and issues. Assessments of climate change impacts vary widely, depending on the situation (e.g., a natural resource/production system such as agriculture, or an economic activity such as investment in infrastructure development); time frame (e.g., near-term consistent with annual crop planning, or longer timeframe comparable to the design lifetime of road transport system); region and area (e.g., a trans boundary watershed or a single site); and purpose of the assessments (e.g., to raise awareness of climate change, or to inform the technical design of large/expensive infrastructure). Furthermore, successful adaptation not only depends on governments, but also on the active and sustained engagement of stakeholders including national, regional, multilateral and international organizations, the public and private sectors, civil society, and other relevant stakeholders (United Nations Framework Convention on Climate Change 2015).

This study described the application of four geodesign tools to support collaborative adaptation planning. The spatial designs that resulted from the use of the tools can contribute to the establishment of a climate resilience society as these local plans can serve as ingredients for adaptation to similar issues at larger scales or in other regions. Both planners and researchers considered the tools developed in this study useful at the scoping stage of an adaptation planning process. The learning by doing aspect of the tools was reflected upon as very effective. The tools were easy to use (Eikelboom and Janssen 2015), and their application positively contributed to extending the knowledge of the participants. The results indicated that the choice for a tool influences the decision-making process as each tool yielded different designs of adaptation measures. The discussions revealed that for each tool, similar logic was used by each group to decide on measures and that the participants tend to cluster changes. Little time was spent on tool interpretation for tools tailored to individual analytical and collective political rationalities, whereas much time was spent on discussing what could be interpreted from the other tools. The application of the individual analytical tool induced many changes while only little time was spent on negotiation of measures. Less changes were made using the remaining tools, where more time was spent on negotiating the allocation of measures. Moreover, the individual analytical tool was preferred by both researchers and planners and was also found to induce many measures in correspondence with tool information. Therefore, we argue that careful selection of methods and tools supports the development of adaptation plans and rationality can be used to choose between different geodesign tools. If the rationality behind the decision process is unclear, the analytical and individual approach would be best as the interpretation and use of this tool was found quick and easy.

Improvements of geodesign tools as well as the users learning process must be seen as an interactive and iterative process. The communication between science and policy can benefit from further tool development by improving user friendliness such as the integration of urban strategy and phoenix (Dias et al. 2013), the inclusion of downscaled climate scenarios, adding information on costs, inclusion of filters to reduce the amount of information in the maps, to add scenarios of measures and improving modeling, and visualization techniques to further tailor tools to specific planning tasks. Further improvements are needed in terms of the availability of the tools to a wider audience (e.g., web tool), and making the tool flexible for different scales and users. Next, the participants emphasized the need for a professional operator and indicated that additional explanation is needed on the exact interpretation of the traffic lights for each type of tool. Furthermore, the tools need constant updates on the latest findings and best available data. To conclude, the development and application of this type of tools is a process rather than a product (Wenkel et al. 2013).

Although the tools were tested to support the design of adaptation plans in a Dutch setting, the tools could be used for regional adaptation planning in other countries such as the development of regional adaptation strategies (RAS) as required by the European Union or on a national scale to support developing National adaptation plans of action (NAPAs) as initiated by the United Nations Framework Convention on Climate Change (UNFCCC) for least developed countries.
